# Ultra-High-Field Magnetic Resonance Spectroscopy in Psychiatry

**DOI:** 10.3389/fpsyt.2017.00123

**Published:** 2017-07-11

**Authors:** Beata R. Godlewska, Stuart Clare, Philip J. Cowen, Uzay E. Emir

**Affiliations:** ^1^Department of Psychiatry, University of Oxford, Warneford Hospital, Oxford, United Kingdom; ^2^Oxford Centre for Functional MRI of the Brain, Nuffield Department of Clinical Neurosciences, University of Oxford, John Radcliffe Hospital, Oxford, United Kingdom

**Keywords:** ultra-high-field, magnetic resonance spectroscopy, psychiatric disorders, neurochemicals, magnetic resonance spectroscopic imaging

## Abstract

The advantages of ultra-high-field (UHF ≥ 7T) MR have been demonstrated in a variety of MR acquisition modalities. Magnetic resonance spectroscopy (MRS) can particularly benefit from substantial gains in signal-to-noise ratio (SNR) and spectral resolution at UHF, enabling the quantification of numerous metabolites, including glutamate, glutamine, glutathione, and γ-aminobutyric acid that are relevant to psychiatric disorders. The aim of this review is to give an overview about the advantages and advances of UHF MRS and its application to psychiatric disorders. In order to provide a practical guide for potential applications of MRS at UHF, a literature review is given, surveying advantages and disadvantages of MRS at UHF. Key concepts, emerging technologies, practical considerations, and applications of UHF MRS are provided. Second, the strength of UHF MRS is demonstrated using some examples of its application in psychiatric disorders.

Psychiatric disorders are related to substantial personal, public, and economic burdens and are responsible for nearly 13% of the global burden of disease in terms of disability-adjusted life years, and a staggering 32% of years lived with disability (1). Multiple lines of research, including brain imaging, analysis of post-mortem brain tissue, and genetic studies have resulted in the identification of potential dysfunctions in psychiatric disorders, including schizophrenia (SCZ) (2), major depressive disorders (MDD) (3), bipolar disorder (BD) (2), anxiety disorders (4), autism spectrum disorder (5), and anorexia nervosa (6). In addition, the development and research application of newer imaging modalities such as mapping the “human connectome” employing magnetic resonance imaging (MRI) is opening new avenues to study brain mechanisms underlying psychological processes non-invasively in the living brain (7).

Most MRI-based imaging modalities (for example, structural imaging) are sensitive to macroscopic alterations. Complementary to MRI, magnetic resonance spectroscopy (MRS) techniques may be utilized to reveal abnormalities before any visible macroscopic changes in brain anatomy and physiology occur, since they provide unique information on the neurochemical composition of the brain tissue (8–10). For instance, neurochemicals that can be measured non-invasively in human brain include (1) endogenous neurotransmitters; glutamate (Glu) and gamma-aminobutyric acid (GABA) (11) (2), psychotropic medications, such as lithium (12) and fluorinated drugs (13). Thus, when applied to *in vivo* brain imaging, MRS can be used to measure the neurochemical composition of brain in order to characterize metabolic processes and identify aberrant neurochemical or metabolic relationships related to psychiatric disorders.

The recent progress in MRI technology such as the use of ultra-high-field (UHF ≥ 7T) magnets, advanced magnetic field (B_0_) shim coils, improved gradient systems, and radio frequency (RF) coils have been enabling robust *in vivo* application of MRS techniques by providing the improved sensitivity and resolution. Specifically, using MRS at UHF, it is possible to measure the signals from 10 to 15 metabolites that might be a marker of different pathophysiological processes of psychiatric disorders (Figure 1) (14). Signal-to-noise ratio (SNR) (defined as peak height divided by root mean square noise) is approximately 1.6 times higher at 7 T relative to 3 T (Figure 2) (15). From 3 T to 7 T, gains in sensitivity are particularly prominent for Glu, glutamine (Gln), and GABA (16). As a result, metabolites are quantified with lower errors (lower Cramér-Rao Lower Bounds) at 7 T than at 3 T, which can be translated to improved quantification metabolite concentrations. Thus, UHF MRS methods have the potential to improve the understanding of the etiology, progression, and the response to therapy in psychiatric disorders due to the improved quantification of metabolites, such as Glu, Gln, and GABA that are relevant to psychiatric disorders.

**Figure 1:**
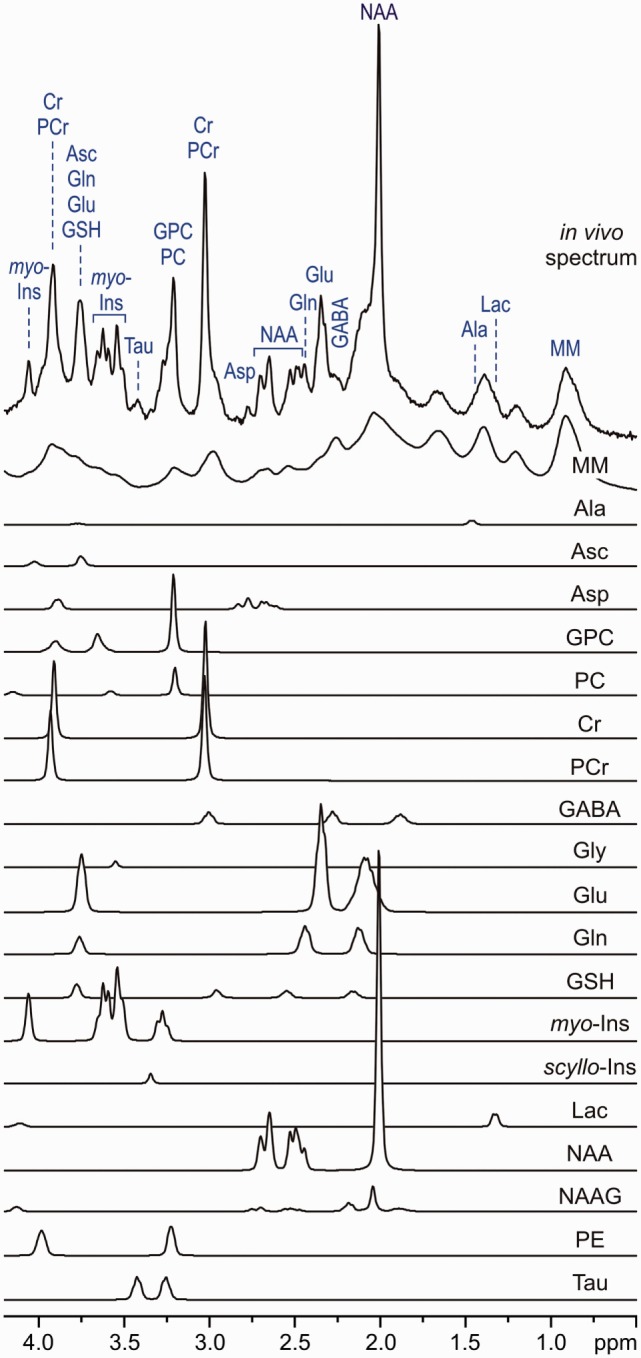
*In vivo* stimulated echo acquisition mode spectrum (volume of interest, 8 ml, TE = 6 ms, TR = 5 s and number of transients, 160) and LCModel fit, modeling metabolite contributions to the neurochemical profile. Model spectra for glycerophosphocholine, phosphocholine, creatine, phosphocreatine, γ-aminobutyric acid, glucose, glutamine, Glutamate, glutathione, lactate, myo-inositol, *N*-acetylaspartate, *N*-acetylaspartylglutamate, scyllo-inositol, and taurine were imported into LCModel (17) and used for spectroscopic quantification [reprint McKay and Tkác (18)].

**Figure 2:**
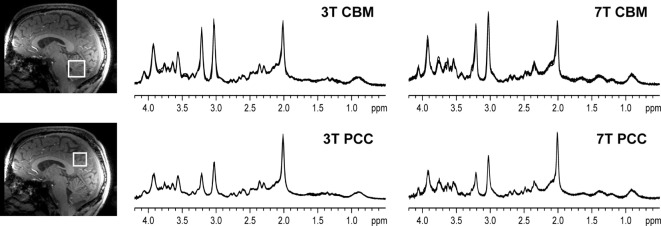
Representative *in vivo* ^1^H magnetic resonance spectroscopy spectra obtained in a healthy volunteer. All spectra obtained in one subject are shown (semi-LASER, TE = 28 ms at 3 T and TE = 26 ms at 7 T, TR = 5 s, 64 transients), with the four spectra obtained per brain region/field overlaid in each panel. The voxel locations are shown on the T_1_-weighted images [reprint Terpstra et al. (16)].

The aim of this review is to give a comprehensive overview of the advantages, challenges, and advances of UHF MRS with regard to methodological development, discoveries, and applications in psychiatric research from its beginnings around 15 years ago up to the current state. At lower magnetic field strengths, MRS has been used *in vivo* for different nuclei, including ^1^H, ^31^P, ^13^C, ^15^N, ^19^F, and ^23^Na; however, psychiatric disorder applications of UHF MRS are limited to ^1^H. For this reason, this review will focus on the use of ^1^H for MRS at 7 T.

## Challenges at UHF

*In vivo* MRS at UHF is ready to make important contributions not only in the evaluation of disease (19) but also more importantly because it may assist to study disease progression and treatment (20, 21). Despite this potential, implementing MRS in the human brain at UHF involves multiple challenges, such as magnetic field and RF inhomogeneity, increased spectral bandwidth and short relaxation times of metabolites.

At UHF, the interaction between the brain tissue and RF pulse results in strong inhomogeneities since the RF wavelength is in the order of human head. For instance, at 7 T, the wavelength of the RF pulse used in the human brain is around 12 cm and generates varying destructive interferences throughout the brain, resulting in RF pulse inhomogeneity. As a result, the insufficient transmit power with conventional volume head coils requires increased RF pulse durations with smaller bandwidths and this in turn introduces a large chemical shift displacement error in the spectra. This problem is worsened in many regions of the brain since volume coils can only provide high enough transmit power in the central region of human head compared with the periphery region (22).

Recent noteworthy investigations demonstrated the feasibility of the use of multiple transmit array coils used to control the distribution of electromagnetic fields and overcome this limitation. It has been demonstrated that such adjusting of RF amplitude and phase to each transmit array coil (RF shimming) results in considerable gains in the efficiency of RF pulses used in single-voxel MRS and magnetic resonance spectroscopic imaging (MRSI) localizations in the brain at 7 T (23). Data quality has been further improved with dynamic RF shimming, especially for MRSI, and several MRSI studies have already demonstrated this approach by utilizing several RF shim settings generated to ensure optimal transmit fields for a VOI-specific homogeneous excitation, a ring-shaped excitation of the skull area for lipid suppression and a global uniform excitation for water suppression (24).

Problems resulting from limitations in available RF magnitude can be partially avoided by utilizing dedicated new excitation and saturation pulses without modifying any hardware at UHF. These pulses should have a high degree of tolerance to RF inhomogeneity with broadband sharp slice-selection profiles. It has been demonstrated that broadband frequency-modulated RF pulses can partially overcome these problem, as in semi-localization by adiabatic selective refocusing (semi-LASER) and localization by adiabatic selective refocusing (LASER) pulse sequences, albeit with a cost of long echo times [50 and 80 ms, respectively (25, 26)]. Alternatively, composite broadband refocusing pulse designs can be used (27). For example, MRSI data can be obtained using the free induction decay acquisition localized by outer volume suppression (FIDLOVS) technique (28), based on 2D pre-localization by outer volume suppression and frequency-modulated excitation pulse for slice-selection.

In addition to RF inhomogenetity, significant main magnetic field (B_0_) inhomogeneity will be introduced as the subject is placed in the scanner due to the magnetic susceptibility of the different tissues. At UHF, the effect is even more marked, since the magnitude of the B_0_ shift is proportional to static field (29). The process of B_0_ shimming usually mitigates these effects. However, for the size of voxels used in single-voxel MRS at UHF, the vendor-provided approaches are not always optimum. At UHF, it is better to use an approach focusing on the volume of interest to minimize strong B_0_ inhomogeneities. One approach is to acquire a B0 field map using two gradient-echo images with different echo times. When choosing the difference in echo time to use for B_0_ shimming, there is a balance between long evolution times giving the best sensitivity and short evolution times giving better signal and avoiding phase wraps. Typically, an evolution time of 2–4 ms is appropriate (30) or it can be better to use multiple evolution times (31). An alternative approach is to use the FASTMAP and its echo-planar-based derivative (32, 33), which measures B_0_ field plots along projections around the voxel of interest. This approach is faster and can be run at a higher resolution than an imaging-based method. For both methods, iterations minimize the B_0_ inhomogeneities.

For MRSI at UHF, where a larger volume of interest needs to be shimmed, it appears to be most beneficial to use higher order (third or fourth) shim terms (34) to achieve narrow linewidths. However, even with these extra terms, it is not possible to completely remove all B_0_ variation. An alternative approach that has shown much promise for MRSI is dynamic B_0_ shimming (35); however, this again requires specialist hardware for the scanner. A number of researchers have shown the benefits of using diamagnetic or paramagnetic passive shims, such as those placed in the mouth (36); however, these approaches are often less comfortable to the subject and require a much more involved optimization procedure. More recently shim coil designs that are not based on spherical harmonics, but that can drive current in an arbitrary pattern, have been proposed (37). These solutions are again technically complex and are some way from being routine.

Transverse relaxation times, T_2_, of metabolites in the human brain decrease as the magnetic field increase (38, 39) and the SNR advantages quickly disappear with increasing echo times. Therefore, short echo times are critical for not only minimizing T_2_ losses and preserving intensity from J-modulation but also when using UHF to study patient populations, who potentially have different metabolite T_2_ values (40).

Recent developments have demonstrated that challenges can be overcome, and the gap between bench and their potential for clinical application can get narrower. As clinical UHF systems become increasingly available, *in vivo* MRS detection of metabolites at UHF benefits from gains in SNR and chemical shift dispersion, which may enable the detection of subtle changes in metabolite levels. MRS allows detection of a variety of metabolites, including *N*-acetylaspartate (NAA) as a marker of neuronal loss/dysfunction, total creatine [tCr, creatine (Cr) + phosphocreatine (PCr)] as a marker for deficits in energy metabolism, total choline (phosphocholine + glycerophosphocholine) as a marker for cell membrane turnover, and Glu and GABA, the primary excitatory and inhibitory brain neurotransmitters, respectively. The improved detection of these metabolites at UHF is of potential value in understanding the neuropathology or biochemical abnormalities in mental health disorders as well as in evaluating disease progression and response to therapeutic interventions. In the following sections, we will review clinically relevant and MRS-detectable metabolites and illustrate potential applications in psychiatric conditions. Furthermore, if applicable, we will provide examples from translational research as they relate to psychiatric disorders.

## Why UHF MRS Can be Useful in Psychiatric Research

As stated above, one of the clear advantages of UHF MRS is an increased SNR, which can allow for a more precise assessment of molecules that would not pass the quality threshold at lower fields due to sensitivity issues. Yet, possibly the greatest advantage for clinical research is the higher spectral resolution that UHF offers and which allows for a more reliable measurement of metabolites that cannot be resolved at lower magnetic fields and are relevant to the pathogenesis of psychiatric symptoms. Up to date, there have only been a few MRS studies at UHF, which we will report in the context of UHF benefits and with a short summary of findings at lower fields.

## Glu, Gln, and GABA

**Figure 3:**
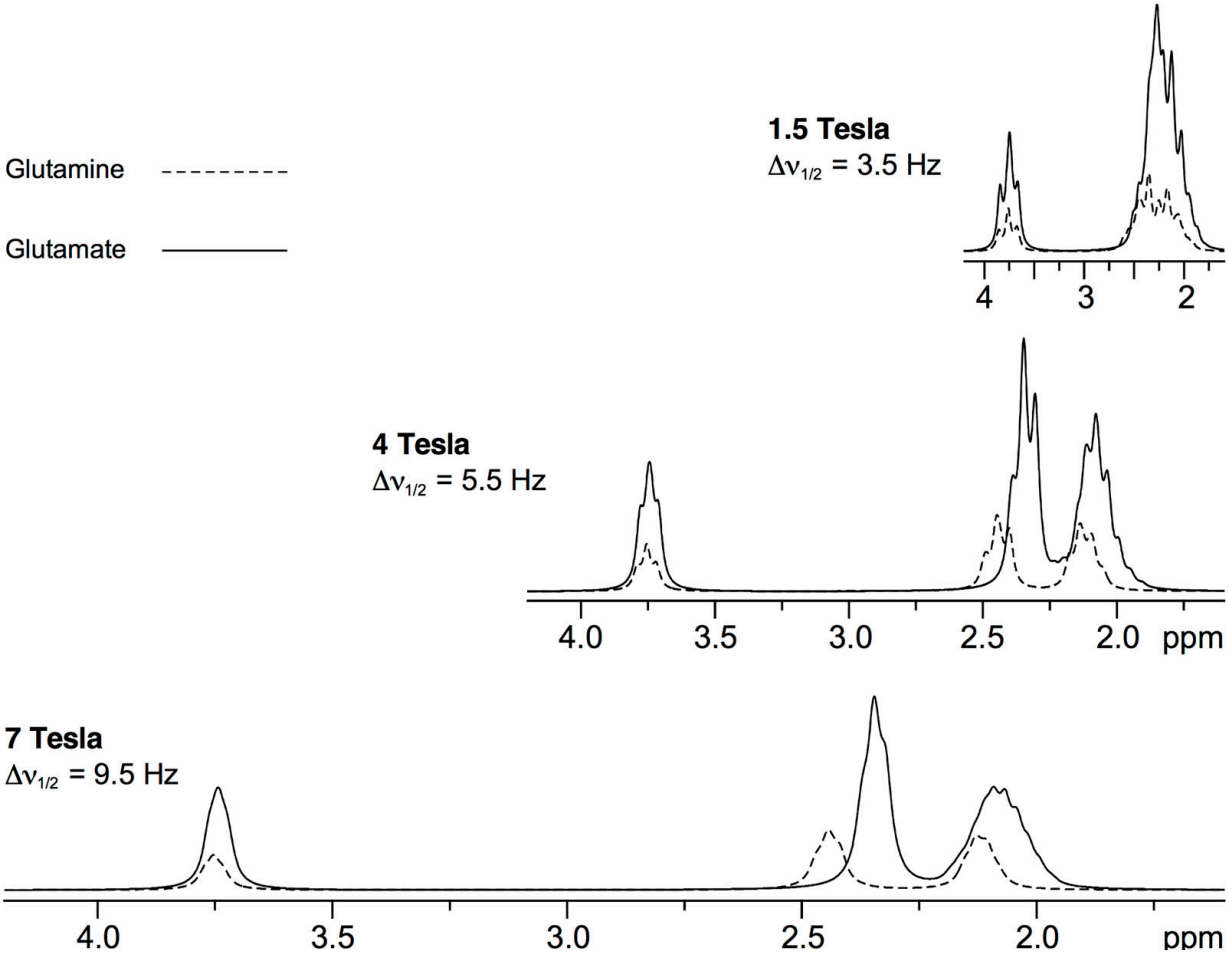
Simulated ^1^H magnetic resonance spectroscopy spectra of glutamate and glutamine at a range of field strengths [reprint Tkác et al. (41)].

Effective separation of Glu and Gln may be one of the most important advantages derived from the use of UHF in the context of psychiatric disorders (41). While the role of Glu and Gln has been postulated in the pathogenesis of major psychiatric conditions, the clear separation of Glu and Gln resonances at UHF compared to lower magnetic fields (Figure 3) might shed light on understanding their plausible mechanisms (41). Glu is the main excitatory neurotransmitter in the brain, present in about 80% of synapses, and it is one of the key components of cellular energy metabolism. It has an average concentration of 6–13 µmol/g with significant differences between gray and white matter (42). Released Glu is taken up into astrocytes where it is converted to Gln. This process prevents the toxic effect of excess Glu on neurons. Subsequently, Gln is released into the extracellular space, to be taken into the presynaptic terminals and converted back into Glu. Gln concentrations are around 2–4 µmol/g (42). GABA, the main inhibitory neurotransmitter, is closely linked to the Glu and Gln molecules. It is produced from Glu by glutamate decarboxylase and has concentration of circa 1–µmol/g (42). GABA plays an important role in maintaining the correct excitation–inhibition balance of cortical networks. Due to its low natural concentrations and proximity of more abundant metabolites of NAA at 2 ppm, tCr at 3 ppm, and Glu and Gln at 2.3 ppm, reliable quantification of GABA benefits from improved SNR and increased resolution offered by UHF compared to lower magnetic fields (43).

To our knowledge, only a few studies have assessed the glutamatergic system at UHF, and most of them focused on SCZ. One study has shown no differences in Glu levels between patients with SCZ and healthy controls in medial prefrontal cortex (mPFC) and parieto-occipital cortex (OCC) (44). There was, however, an effect of age and gender on mPFC Glu concentration, with lower Glu in older participants and in males, irrespective of disease. These are also found lower GABA/Cr ratio in patients in mPFC but not in parieto-OCC.

Another 7 T study explored differences between medicated patients with SCZ, their healthy relatives and healthy non-related controls – finding reduced cortical Glu in patients with SCZ compared to healthy controls, as well as reduced Glu in healthy relatives, suggesting that Glu concentrations were associated with genetic load for illness (45). They also found reduced cortical GABA in patients compared with both healthy relatives and the pooled sample of healthy individuals (relatives and non-relatives), suggesting an association of altered GABA concentrations in SCZ with either illness state or medication effects. There were no differences in metabolite concentrations in basal ganglia.

One other 7 T study has showed an effect of age in relation to illness, with higher Glu levels in ACC in patients with SCZ under the age of 40 compared to controls, with no differences between patients over 40 and controls (46). An inverse correlation of Glu with age was found for patients but not controls, suggesting an ongoing pathological process during which Glu might decline through the course of the disease. There were no differences in Glu, Gln, Glu/Gln ratio, or GABA when comparing all patients to all controls.

To our knowledge, there has been only one UHF study in mood disorders—a 7 T MRSI investigation in MDD, which showed no statistically significant difference in Glu, Gln, and GABA levels across the number of regions, although there was an increase in Glu after 8 weeks of mindfulness-based cognitive therapy (MBCT) (47).

A pilot 7 T study has shown widespread reductions in Glu in anorexia patients as compared to healthy controls (48). Previous studies at 3 T in this group of patients measured Glx (a combined measure of Glu and Gln), and some, although not all of them, reporting a decrease. A separation enabled by UHF MRS allowed for the identification of the likely key contributor to this change.

Complementary to the fMRI, functional MRS (fMRS) could help relate functional impairments to psychiatric disorder. The potential of fMRS has been demonstrated by detecting metabolic changes in the brain induced by different types of physiological interventions (49–52). For instance, a recent study has demonstrated a correlation between Glu and BOLD-fMRI time courses using a novel combined fMRI-MRS sequence (53). Thus, fMRS could be used to measure neurochemical levels to study glutamatergic response and/or energy metabolism in psychiatric disorders during different physiological interventions. A recent fMRS study at 7 T has successfully demonstrated differential response of the glutamatergic system to a Stroop task in patients with SCZ, MDD, and healthy controls (54).

Ultra-high-field studies are a potentially useful tool in drug discovery. They can help to understand more precisely mechanisms of action of medications and explore their potential in the context of known pathophysiology of psychiatric conditions. This would apply to both existing treatments, with the view of repurposing, and new ones, whose mode of action may suggest their potential use in known pathologies. Two UHF studies on medication effect can serve as a good example. One of them studied the effect of gabapentin, indicated for treatment of focal seizures, peripheral neuropathic pain, and migraine prophylaxis, which has also been used as an adjunctive treatment in mood disorders. In a 7 T study Cai et al. (20) found a mean 55.7% increase in occipital GABA in response to gabapentin challenge in healthy volunteers. This was consistent with another study in healthy volunteers performed at 4.1 T (55). Given that a decrease in GABA in MDD has been postulated (56), this effect, although obtained in healthy volunteers, is of potential interest to MDD treatment. Another study in healthy participants has shown a decrease in Glu and Gln under the influence of ebselen, a potential lithium-mimetic and glutaminase inhibitor, in healthy volunteers (57). This finding supports its further exploration as treatment for BD in the light of postulated increased in Glx levels in this condition (the effect of ebselen on *myo-*inositol (*myo*-Ins) is described below).

These findings at UHF would be important for interpretation of lower magnetic field studies in psychiatric disorders where there are many conflicting results, partly because distinguishing Glu, Gln, and GABA is difficult due to the lower SNR and spectral resolution. For instance, a meta-analysis by Merritt et al. (58) in SCZ suggested elevations in glutamatergic metabolites across several brain regions, including limbic areas and frontal regions. Another meta-analysis (59) suggested reduced Glu and elevated Gln in frontal regions in patients, with their levels decreasing with age but only in the patient group. A review by Poels et al. (60) suggested that Glu activity (as indexed by Glu and Glx levels) might be elevated in medication-free early-stage patients with psychosis, while they decrease after treatment and in chronic patients.

Again at conventional magnetic fields, GABA-related findings in SCZ show increase, decrease, and no difference (61). One meta-analysis showed a trend toward lower GABA in patients, which, however, did not reach significance (56). This, however, would be consistent with two of the 7 T studies in SCZ noted above (44, 45).

Findings from 3 T studies suggest, to a reasonably consistent degree, decreased Glu and Glx levels in MDD and increased levels in BD, as suggested by meta-analyses (62–64) and reviews (65–68). The changes were shown across a number of regions important for mood processing, such as prefrontal cortex, the hippocampus and the amygdala, across different ages (69), and, in BD, seemed to be independent of the current mood state or medication status. Only a few studies attempted discrimination between Glu, Gln, and GABA at 3 T using J-resolved (70) and J-editing MRS sequences (71) in depressed patients. Both a decrease (71) and no change in GABA (70) levels were reported. A recent meta-analysis (56) has shown reduced GABA concentration in currently depressed MDD patients but not in remitted MDD patients compared with healthy controls, while there were no differences between patients with BD and controls.

## NAA and *N*-Acetylaspartylglutamic Acid (NAAG)

Another two molecules that can benefit from increased separation at UHF are NAA and NAAG (15). NAA is mainly found in neurons, it is considered a marker of neuronal integrity/function, and its concentration is in the range of 8–14 µmol/g under normal conditions (42). NAAG is a derivate of NAA and Glu and acts as an agonist at metabotropic Glu receptor type 3 (72). Animal studies indicate that it may play a role in psychiatric conditions. For instance, it has been demonstrated that an alteration of synaptic NAAG levels represents a new therapeutic approach to treating the positive and negative symptoms of SCZ (73). Rare reports on NAAG encourage further exploration, showing differences in its concentration between patients with SCZ and healthy controls (74). The singlet of NAA at 2.02 ppm is the most prominent metabolite signal, whereas the detection of NAAG in human brain by MRS is challenging at lower magnetic field due to its relatively low concentration and the overlap with intense signals of NAA and Glu. Similar to the separation of Glu and Gln at UHF, the separation of NAA and NAAG also benefits from improved resolution and increased SNR at UHF. Interestingly, a recent 7 T study has shown a reduction in NAA/tCr levels in patients with MDD, which normalized after 8 weeks of MBCT treatment (47).

Large meta-analyses of lower field studies in SCZ, reported possibly disease stage-related NAA reductions in frontal and medial temporal regions (75, 76) and in basal ganglia (75). In BD, a large meta-analysis (76) found an NAA decrease in basal ganglia only, while there were elevations in dorso-lateral prefrontal cortex; in this second region, data heterogeneity across the included studies was high, however. It was suggested that in BD, medication status, particularly with lithium, might be a confounder. Indeed, some studies showed a decrease in NAA in drug-free BD patients, with some of NAA increases after treatment with lithium (63). There is no convincing evidence of NAA changes in MDD (62, 69, 77).

## Cr and PCr

Total Cr, a combined measure of Cr and PCr (tCr, Cr + PCr), is often used as a reference molecule in MRS analysis. However, tCr level should be used with caution as an internal concentration reference since it might change with disease, complicating the interpretation of changes in ratios relative to tCr (78, 79). Cr and PCr have an important physiological role acting as a system for quick generation and storage of energy by moving phosphate groups between ATP and ADP in anaerobic processes in tissues and organs with high and quickly changing energy demands, such as the brain. Changes in their concentration may be linked to disturbances in energy metabolism and their assessment can provide important information about underlying pathologies. Most studies assessing PCr-Cr cycle used ^31^P MRS, with changes reported in a number of psychiatric conditions, such as SCZ (80). While this is an informative way of assessing PCr-Cr cycle activity, information on other metabolites of interest cannot be acquired simultaneously, which would be the main benefit of UHF in this context (81). A discussion of 31 P studies is beyond the scope of this paper.

## *myo*-Ins and Glycine (Gly)

The main, resolved resonance of *myo*-Ins is at 3.56 ppm (42). This resonance, at lower magnetic field strength also contains contributions from the amino acid, Gly (42). *myo*-Ins is one of the larger signals in short echo time spectra, with a concentration of 5–10 µmol/g whereas Gly present in normal human brain at up to a 1 µmol/g concentration (42). *myo*-Ins is a precursor for the phosphatidylinositol second messenger system. Predominantly located in astrocytes, it is often considered to be a glial marker. It also has an established role in osmoregulation. Gly is a co-agonist of Glu for the NMDA receptor and is necessary for Glu effect. Due to this, it may play an important role in the pathogenesis of disorders for which dysfunction of the glutamatergic system has been postulated, such as mood disorders and SCZ. In addition, it has been shown that Gly administration to SCZ patients improves the efficacy of conventional antipsychotic drugs, such as olanzapine and risperidone (82).

At lower magnetic fields, Gly detection requires specific MRS sequences to separate *myo*-Ins and Gly signals, such as the two-dimensional J-PRESS (83) and TE-optimized triple refocusing (84). At UHF, the improvement in separation of *myo*-Ins and Gly resonances has been demonstrated without requiring any specific MRS sequence (85). Thus, the use of UHF creates a unique opportunity for the separation of these two molecules, a finding potentially important for treatment development.

As for *myo*-Ins, two recent 7 T studies have reported decreased levels of *myo*-Ins in MDD, one in the insula (47), with levels correlating with depression severity as measured by Hamilton Depression Rating Scale (HAMD-17), and one in ACC and thalamus (86). Another 7 T study has shown a reduction in *myo*-Ins in the ACC and OCC of patients with anorexia nervosa (48). Two studies in patients with SCZ that reported *myo*-Ins found no differences in ACC (44) and thalamus (86) between patients and controls. As for Gly, a recent 7 T study (86) found reductions in the thalamus but not ACC of patients with SCZ as compared to healthy controls and patients with MDD.

*myo-*inositol is perhaps most interesting in the therapeutic context, given that lithium, an effective mood stabilizer, has an ability to diminish its levels and potentially thereby lower signaling through synapses employing the PI cycle as a second messenger system. A 3 T study in healthy volunteers aimed to assess the effect of ebselen, a putative lithium-mimetic and IMPase inhibitor and found a decrease in ACC *myo*-Ins under its influence (78); the finding was then replicated on a 7 T scanner (57), with the additional benefit of providing information on Glu and Gln changes, described above.

Only a few studies at lower fields showed differences in *myo*-Ins levels between patient populations and healthy controls, e.g. decreased *myo*-Ins in frontal areas of the brain in MDD (87). However, due to signal overlap between *myo*-Ins and Gly, Gly has not been reported in low-field MRS studies of psychiatric disorders.

## Glutathione (GSH)

The importance of GSH relates to its role as a major endogenous antioxidant removing free radicals and, hence, protecting cells against the effect of oxidative stress. GSH peaks appear as a singlet at 3.77 ppm, multiplets at 2.15 and 2.55 ppm, and doublet of doublets at 2.93, 2.98, and 4.56 ppm (42). It has relatively high concentrations in the brain (2–3 µmol/g) (42). However, it is challenging to measure *in vivo* due to significant resonance overlap with other metabolites. GSH can be detected using selective editing techniques at lower magnetic fields. In addition, a reasonably robust measurement from non-edited short echo spectra using fitting routines such as LCModel has been demonstrated (88). Recent MRS studies without special editing techniques at UHF demonstrated improved GSH quantification providing valuable new insights into physiatrist disorders (89).

The role of increased oxidative stress has been postulated in the pathogenesis of major psychiatric conditions. Measurement of GSH is challenging at lower fields and it was reported in only a few 3 T studies (90, 91). MRS at UHF improves GSH quantification providing (89). A 7 T study in MDD observed a decrease in GSH in the putamen, in line with the findings of a 3 T study (47), which has shown a decrease in the OCC of MDD patients. The aforementioned 7 T study on the effect of ebselen has shown a decrease in GSH under the influence of the drug (57). Two other 7 T studies, one in SCZ (46) and one in anorexia (48) did not observe any differences between patients and healthy controls.

## Limitations and Potential

Scanning at UHF, although in many ways advantageous, is not free from limitations, which can hamper its use for clinical applications. One such limitation is potential safety hazards. While no physiological health hazards have been identified in connection to UHF, there are potential dangers related to higher magnetic fields, such as the potential for tissue heating, and effects of UHF on implanted devices, such as surgical clips, coils, and stimulation effects. More research is underway in the centers across the world to assess dangers related to specific devices. In practical terms, however, safety protocols lead to a significant proportion of patients being excluded from studies, which may make results from 7 T investigations difficult to generalize. Another issue is the cost of scanning, which is likely to change with time but currently can make clinical applications impractical. Last but not least, UHF studies share some of the limitations with lower field studies. Such limitations include, among others, differences between volunteers in different studies, the lack of clinically relevant stratification, for instance in terms of disorder severity or stage of the condition, medication status, differences in study design or MRS signal acquisition, and post processing, which can make results difficult to compare. The emerging, albeit at this point limited, picture of UHF studies suggests that they may not be free from inconsistencies characterizing lower field studies.

Despite these limitations, UHF MRS may offer important advantages over lower fields, thanks to which it could become a useful and powerful tool in the clinical context. It can provide information about molecules crucial to both physiological functions of the brain and pathogenesis of psychiatric conditions, assessment of which could not be reliably performed at lower fields. In the future, UHF MRS may explore approaches based on other nuclei, such as ^31^P, which will increase the amount of information that can be obtained. Importantly, UHF MRS may also be useful as a tool for drug discovery in terms of both understanding existing treatments and testing the neurochemical effects of novel pharmacological approaches.

## Author Contributions

Conception and design: UE and BG. Writing, review, and/or revision of the manuscript: UE, BG, PC, and SC.

## Conflict of Interest Statement

The authors declare that the research was conducted in the absence of any commercial or financial relationships that could be construed as a potential conflict of interest.
